# Archetypes of Binocular Visual Field Loss and Their Impact on Vision-Related Quality of Life in Glaucoma Patients

**DOI:** 10.1167/iovs.67.3.28

**Published:** 2026-03-12

**Authors:** Mehrdad Gazanchian, Ashkan Nejad, David P. Crabb, Giovanni Montesano, Nomdo M. Jansonius

**Affiliations:** 1 Department of Ophthalmology, University Medical Center Groningen, University of Groningen, The Netherlands; 2 Department of Research and Improvement of Care, Royal Dutch Visio, Huizen, The Netherlands; 3 Department of Optometry and Visual Science, City, University of London, London, United Kingdom

**Keywords:** glaucoma, visual field (VF) defect, archetypes, quality of life (QoL)

## Abstract

**Purpose:**

Binocular visual field (VF) loss affects vision-related quality of life (VR-QoL) but the relationship between specific VF loss patterns and difficulties with specific tasks remains poorly understood. Archetypal analysis offers a promising method to elucidate spatial patterns of VF loss. In this study, we aimed to develop archetypes for binocular VF loss and assess their relationship with VR-QoL.

**Methods:**

We included 7305 pairs of reliable standard automated perimetry (24-2 SITA fast and standard) test results of patients from 5 glaucoma clinics in England from a Healthcare Quality Improvement Partnership-funded audit. We executed an archetypal analysis on the corresponding integrated VFs (estimates of the binocular VFs from pairs of monocular VFs) to identify the best-fitting set of archetypes from our dataset. Then, we used these archetypes to deconstruct the binocular VF of 269 patients with glaucoma from the Netherlands that had completed 4 different VR-QoL questionnaires. Finally, relationships between each of the archetypes and different aspects of VR-QoL were analyzed.

**Results:**

The archetypal analysis demonstrated that a solution comprising 12 archetypes provides the best-fitting model. Our analysis of the 269 patients’ with glaucoma data showed various significant and plausible relationships between the different archetypes and various aspects of VR-QoL.

**Conclusions:**

Our results demonstrate how archetypes can elucidate relationships between the location of a VF defect and different aspects of VR-QoL in glaucoma. This may help clinicians and patients better understand the impact of different types of VF defects.

Glaucoma is a chronic optic neuropathy with generally irreversible damage, although limited functional recovery may occur in some cases.^1^ This damage leads to visual field (VF) loss and may result in blindness if left untreated. Glaucoma significantly impacts patients’ vision-related quality of life (VR-QoL), as the progressive VF loss can hinder daily activities, mobility, and emotional well-being.^2^ Assessing the impact of glaucoma on VR-QoL is crucial for understanding the disease burden and making informed treatment decisions.

Previous research has shown that the binocular VF is a better predictor of patients’ VR-QoL, therefore investigating the relationship between different patterns of binocular VF (BVF) defects and various aspects of VR-QoL is essential for understanding the multifaceted impact of glaucoma on patients’ lives.^3^ Earlier studies, using a variety of analysis methods, showed that specific patterns of VF loss correspond to impairments in different aspects of VR-QoL.^4^^–^^9^ However, the results of these studies are ambiguous. For instance, Sawada et al. and Murata et al. both highlighted that central VF defects have a more significant impact on VR-QoL than peripheral defects,^4^^,^^6^^,^^7^ whereas Peters et al. showed that defects in the 4 most central points in the 24-2 visual field are not independently associated with a lower VR-QoL,^9^ which we confirmed recently.^10^ Although several studies found that inferior hemifield defects are more detrimental to VR-QoL than superior hemifield defects, the impact of defect location can vary depending on the specific task,^4^^,^^6^^,^^11^^–^^14^ and this issue has not been sufficiently studied. By elucidating the link between different patterns of BVF defects and different aspects of VR-QoL, clinicians can better predict the functional implications of glaucomatous damage and tailor treatment and rehabilitation strategies accordingly.

Archetypal analysis, a statistical method for identifying prototypical patterns within complex datasets, is a promising method to elucidate spatial patterns of VF loss. This unsupervised machine learning technique has been used to quantify patterns of monocular VF loss in glaucoma, optic neuritis, and idiopathic intracranial hypertension.^15^^–^^17^ However, no study has yet examined archetypes for BVF loss in glaucoma.

This research aims to advance our understanding of the complex interplay between glaucomatous VF loss and VR-QoL. For this purpose, we (1) identified the BVF loss archetypes of glaucoma, using a large VF dataset from the United Kingdom, and (2) determined the associations between the identified archetypes and different aspects of VR-QoL, using an independent dataset from the Netherlands.

## Methods

### Patient Inclusion

We identified BVF loss archetypes by analyzing a dataset from the Healthcare Quality Improvement Partnership commissioned National Ophthalmology Database Audit, which included 6,024,329 VF records from people attending 5 regionally different glaucoma clinics in England from 2000 to 2015.^18^ Henceforth, we refer to this dataset as the NOD-UK dataset. For our study, we included bilateral pairs of Humphrey field analyzer (HFA) 24-2 SITA fast and standard VF tests performed during the same visit. VF tests that had (1) false positives greater than 10% or (2) false negatives greater than 10% with simultaneously fixation losses exceeding 20% were excluded from the study. We combined the false negative results and fixation losses because, compared with the false positive results, they are considered less significant as reliability metrics.^19^^,^^20^ To mitigate the learning effect associated with VF testing, patients with only one reliable HFA test were excluded. In case of more than one reliable VF, the most recent one was included in our study. Although learning sometimes continues after the first VF,^19^^,^^21^^,^^22^ by far the largest effect occurs between the first and second VFs, indicating that it should be possible to get a useful estimate of the actual pattern of the VF loss as of the second VF.^23^ In order to make sure that we excluded patients with neurological defects (as we aimed to include only patients with glaucoma), we used the method introduced by Kihara et al.^24^ In this method, each VF pair is represented as a dot on a plot where the *x* coordinate is the vertical VF asymmetry and the *y* coordinate is the interocular correlation. Vertical VF asymmetry was calculated as the mean of the absolute pointwise differences in sensitivity (raw decibel [dB] values) between mirror locations across the vertical midline in one VF, and interocular correlation was defined as the correlation between corresponding points (excluding the 2 nasal locations) of each VF pair. Patients who have a high vertical asymmetry and high interocular correlation will form a cluster and these patients may have a post-chiasmatic neurological defect. Authors N.M.J. and M.G. evaluated the VFs of these patients and removed those who had neurological defects. This resulted in the exclusion of an additional 53 patients. Finally, our archetype analysis was based on a sample size comprising the BVFs of 7305 patients with glaucoma.

We used the identified BVF loss archetypes to assess the relationship between different aspects of VR-QoL and BVF loss. For this, we used a dataset of 269 patients with glaucoma from the Groningen Longitudinal Glaucoma Study (GLGS). The GLGS is an ongoing collection of diagnostic glaucoma data collected during regular care at the ophthalmic outpatient department of the University Medical Center Groningen, The Netherlands, begun in 2000.^25^ For the current study, we required the presence of reliable standard automated perimetry (SAP) VFs; no further exclusion criteria were applied in order to obtain a representative sample of clinical glaucoma cases. The GLGS includes both patients with glaucoma and suspects. For this study, only patients with glaucoma were considered. For this, the glaucoma hemifield test had to be outside normal limits in at least one eye on the two most recent VFs. The VF defects had to be in the same hemifield with at least one depressed test point in the same location on both fields, compatible with glaucoma, and without any other explanation. We applied the same reliability criteria as used for the NOD-UK dataset and we excluded similarly those who had only one reliable HFA test. From the patients with glaucoma in the GLGS, 269 patients were selected because they had answered 4 different VR-QoL questionnaires as part of an earlier study.^25^^,^^26^ Visual field data used were from HFA tests performed no longer than 1 year before the questionnaire was administered. We refer to this database as the Netherlands database, hereafter.

### Integrated Visual Field Calculations

The BVF was quantified using the integrated visual field (IVF) concept.^27^ The IVF was calculated for each patient by integrating the right and left monocular VFs using the binocular summation method as described by Nelson-Quigg et al.^28^ The pointwise binocular visual field sensitivity (*B*) is calculated using the formula:

(1) $$\begin{array}{c} B\;=\;\sqrt{R^{2}+\;L^{2}} \end{array}$$

where *R* is the pointwise sensitivity (raw dB value) from the right eye and *L* the pointwise sensitivity from the left eye. We then defined IVF MS (mean sensitivity) as the mean of pointwise BVF sensitivity.

### Archetypal Analysis

We used the method described by Elze et al. to perform the archetypal analysis.^15^ To determine the best-fitting set of archetypes, we tested the performance of the archetypal analysis in predicting the data using different numbers of archetypes, from 2 to 17. To achieve this, we implemented a 10-fold cross-validation method. We randomly divided the IVF data into 10 separate folds. Nine folds were used for training to extract the archetypes, whereas one-fold was used for validating the archetype model performance. We repeated the extraction of the archetype model from a single training set 100 times and selected the archetype model with the best performance (lowest residual sum of squares) on that training set. We then used this best archetype model to linearly approximate the IVFs in the validation fold and the archetypes’ model performance was quantified with the residual sum of squares (RSS). This process was repeated 10 times, each time isolating different folds for validation and training. This whole process was repeated for each number of archetypes from 2 to 17. As a result of the 10-fold cross-validation, we had 10 RSS values for each number of possible archetypes.^15^ Similar to Elze et al.,^15^ the Bayesian Information Criterion (BIC) was implemented to find the best-fitting number of archetypes, which is the number of archetypes that had the lowest error while preventing overfitting, based on the acquired RSS values. A shiny app for decomposing a VF of an individual patient into archetypes using the archetypal model is publicly available at: https://8z90er-mehrdad-gazanchian.shinyapps.io/archetypes-app.

### Questionnaire Data

We utilized a questionnaire compiled from 4 existing sources: (1) the National Eye Institute Visual Function Questionnaire (NEI-VFQ-25),^29^ (2) the NEI-VFQ-25 Neuro-Ophthalmology Supplement,^30^ (3) the Glaucoma Quality of Life-15 Questionnaire (GQL-15),^31^ and (4) a luminance-specific questionnaire developed by Bierings et al.^32^ To minimize the number of questions participants needed to answer and to eliminate redundancy, we selected 43 questions from these sources. Questions that were repetitive, highly similar, or focused on the psychological impact of glaucoma (vision-specific mental health, role difficulty, and dependency subscales of NEI-VFQ 25) were excluded. The questionnaire used is available as Supplementary Material. Depending on their communication preference recorded in the hospital files, participants received the questionnaire either via email or post. All responses were graded using the NEI-VFQ-25 scoring system, where “no problems” received the highest score and “many problems” the lowest. Hence, higher scores correspond to a better QoL. NEI-VFQ-25 subscale scores (subscales are groups of related questions, which reflect specific aspects of VR-QoL including driving, near activities, distant activities, and social functioning) and individual question scores were standardized on a scale of 0 to 100. Subscale scores were calculated using the standard NEI-VFQ-25 scoring algorithm applied to the retained NEI VFQ-25 questions.

### Statistical Analysis

All statistical analysis was performed using *R* software and the *Archetypes R* library.^33^ Ordinal logistic regression was used to examine the relationship between the identified BVF loss archetypes and various aspects of VR-QoL. We built separate models for each aspect of VR-QoL (subscale or individual question). Each model had the concerning aspect as the dependent variable and all archetypes (except the normal archetype) as the independent variables. Models were adjusted for age, gender, and binocular foveal sensitivity (calculated using quadratic summation; Equation 1). Due to the high correlation between IVF MS and the weights of archetype 1 (the archetype corresponding to a normal BVF), 12, and the sum of all archetypes except archetype 1, IVF MS was not included as a covariate in the model to avoid multicollinearity. Moreover, we did not include archetype 1 in the ordinal regression, as this archetype acts as the reference (and is thus part of the intercept). The sum of the weights of the included archetypes is always 100%. To adjust for multiple comparisons, the Bonferroni method was applied to the whole ensemble of questions (subscales for NEI-VFQ-25; individual questions for the other questionnaires) and not in a questionnaire-by-questionnaire manner, setting significance at 0.05/43.

## Results

The Table shows the characteristics of our sample populations. The UK-NOD dataset was used to determine the best-fitting BVF loss archetype structure; the Netherlands dataset, which consisted of 269 patients with glaucoma (64% with bilateral glaucoma), was used to relate the archetype structure with VR-QoL.

**Table.** Characteristics^*^ of the Study Populations

	UK-NOD Dataset (*n* = 7305)	Netherlands Dataset (*n* = 269)
Female sex, *n* (%)	4018 (55)	117 (43)
Age, y	77 (69 to 83)	72 (57 to 80)
Better eye VA, logMAR	0.16 (0 to 0.2)	0.00 (−0.18 to 0.51)
Worse eye VA, logMAR	0.2 (0.06 to 0.3)	0.25 (0.00 to 1.74)
Better eye MD, dB	−2.35 (−4.83 to −0.86)	−4.0 (−13.4 to −0.8)
Worse eye MD, dB	−5.26 (−11.94 to −2.47)	−13.0 (−23.0 to −5.0)

MD, standard automated perimetry mean deviation; VA, visual acuity.

* = Median with inter-quartile range unless stated otherwise.

**Figure 1.**
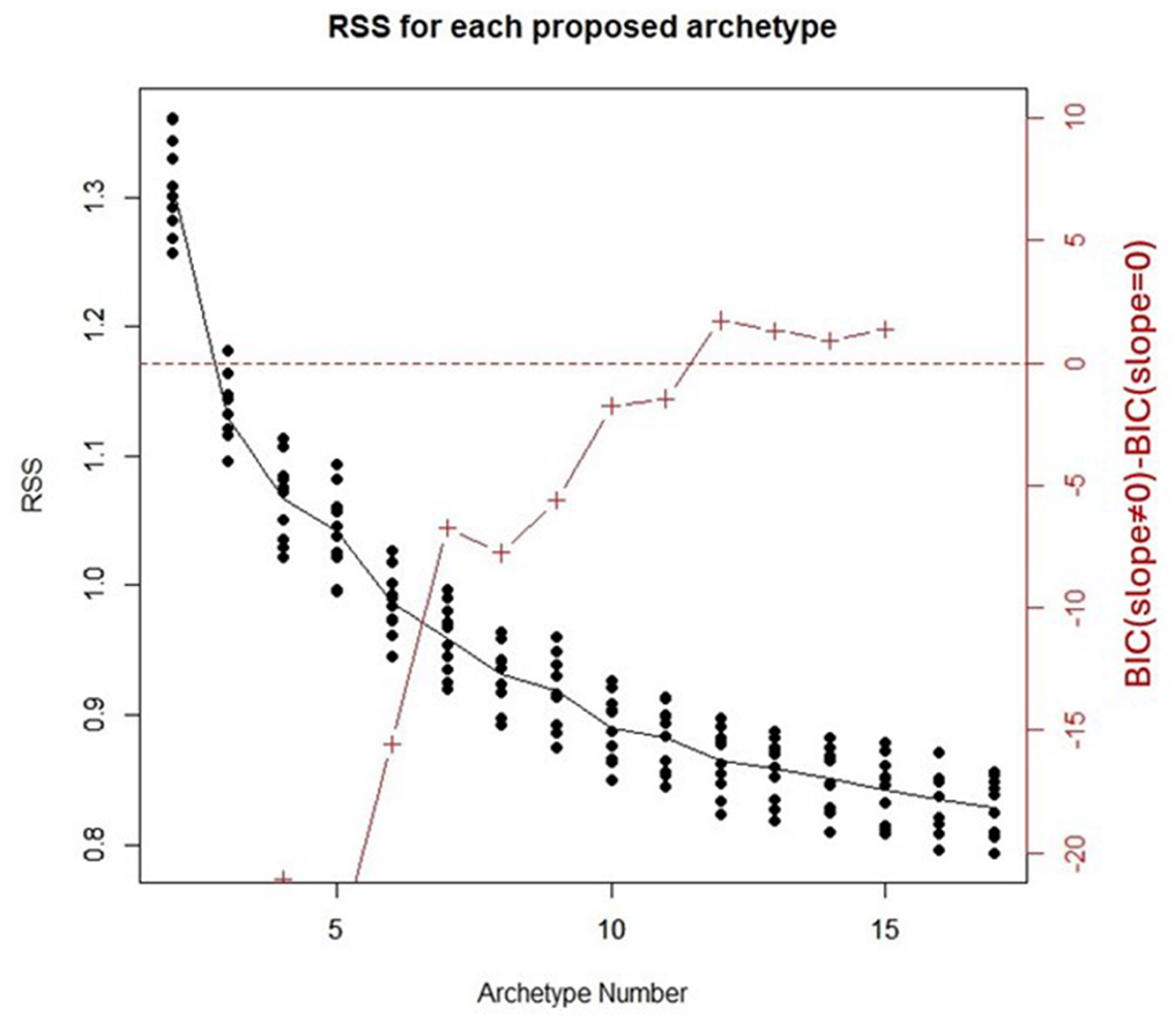
Residual sum of squares (RSS) value of the results of 10-fold cross-validation. Each *black dot* is the RSS value of each fold in that proposed number of archetypes. *Dots* in each archetype number are connected by their median. *Red crosses* represent the difference between the Bayesian Information Criterion (BIC) of the model with a slope ≠ 0 and a null model with slope = 0. A positive BIC difference indicates the best-fitting number of archetypes.

Figure 1 shows the RSS values as a function of the number of archetypes, for each of the 10 folds used as the validation set. The BIC criterion showed that 12 is the best-fitting number of archetypes for decomposing our data.

**Figure 2.**
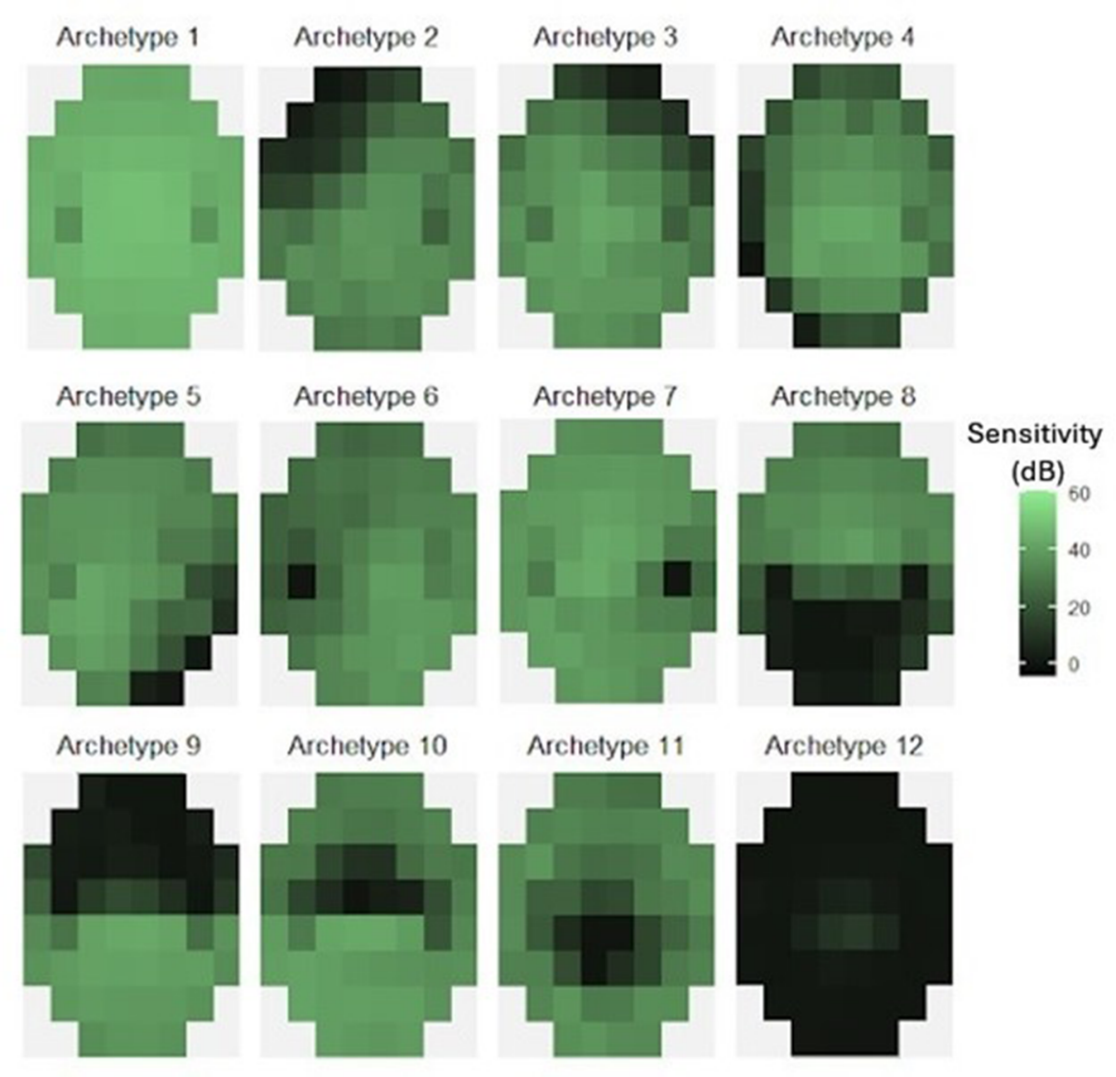
The 12 archetypes of binocular visual field loss in glaucoma.

Figure 2 shows the 12 archetypes. Archetype 1 represents a normal BVF. Archetypes 6 and 7 represent a defect in the blindspot location from the right and left eyes, respectively. Archetypes 8 and 9 correspond to defects in the inferior and superior hemifield, respectively. Archetypes 10 and 11 correspond to defects in the superior and inferior paracentral region of the BVF, respectively.

**Figure 3.**
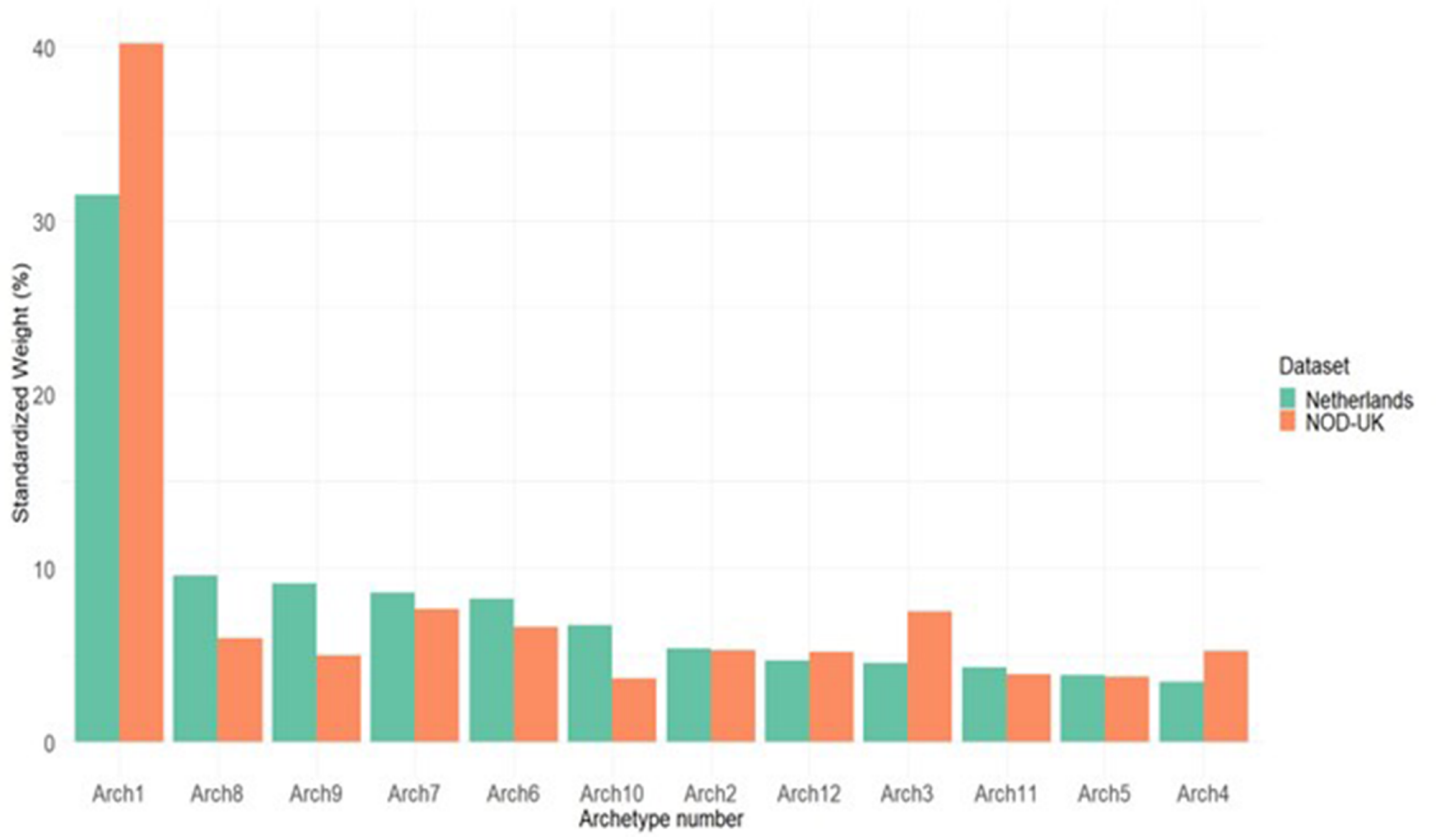
Standardized weight of the 12 archetypes of binocular visual field loss in glaucoma in the NOD-UK dataset, which was used for defining the archetypes, and the Netherlands dataset.

Figure 3 shows the distribution of the different archetypes in the two datasets that we used in this study. Archetype 1 is the most common archetype in both datasets. The other archetypes showed lower frequencies, which were similar within and between the datasets.

**Figure 4.**
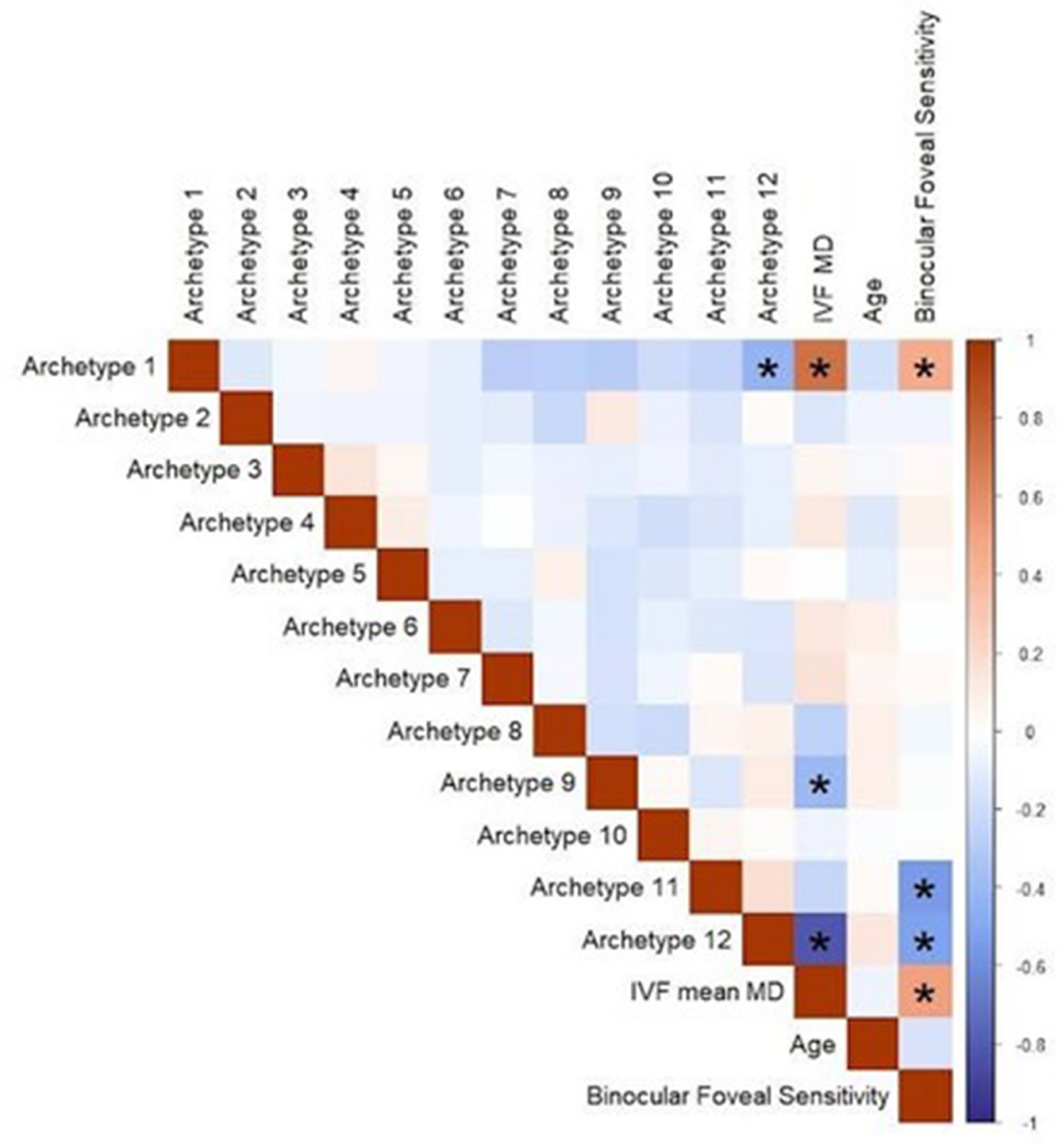
The correlation between different archetypes and clinical attributes of patients. *Asterisks* denote statistically significant correlations using Bonferroni adjusted thresholds.

After defining the BVF loss archetypes, we sought to analyze the relationship between different aspects of VR-QoL and the different archetypes – using the Netherlands dataset. We first carried out a correlation analysis between the archetypes and clinical attributes of the included patients. As shown in Figure 4, archetypes did not correlate significantly with each other, except for archetype 12 (the general loss of sensitivity archetype) and archetype 1 (the normal archetype). Binocular foveal sensitivity had a significant correlation with archetypes 1, 11, and 12, and IVF MS. However, the correlations were modest and unlikely to cause multicollinearity. IVF MS had a significant correlation with binocular foveal sensitivity and archetypes 1, 9, and 12, which were especially strong for archetypes 1 and 12. Therefore, we did not include IVF MS in our ordinal logistic regression analysis. Because the sum of all archetypes for each participant is always 100%, including all archetypes, would result in overdetermination. Therefore, archetype 1, the normal archetype, was excluded; indirectly, it is represented by the intercept, and as such it is used as the reference category for comparison with the other archetypes.

**Figure 5.**
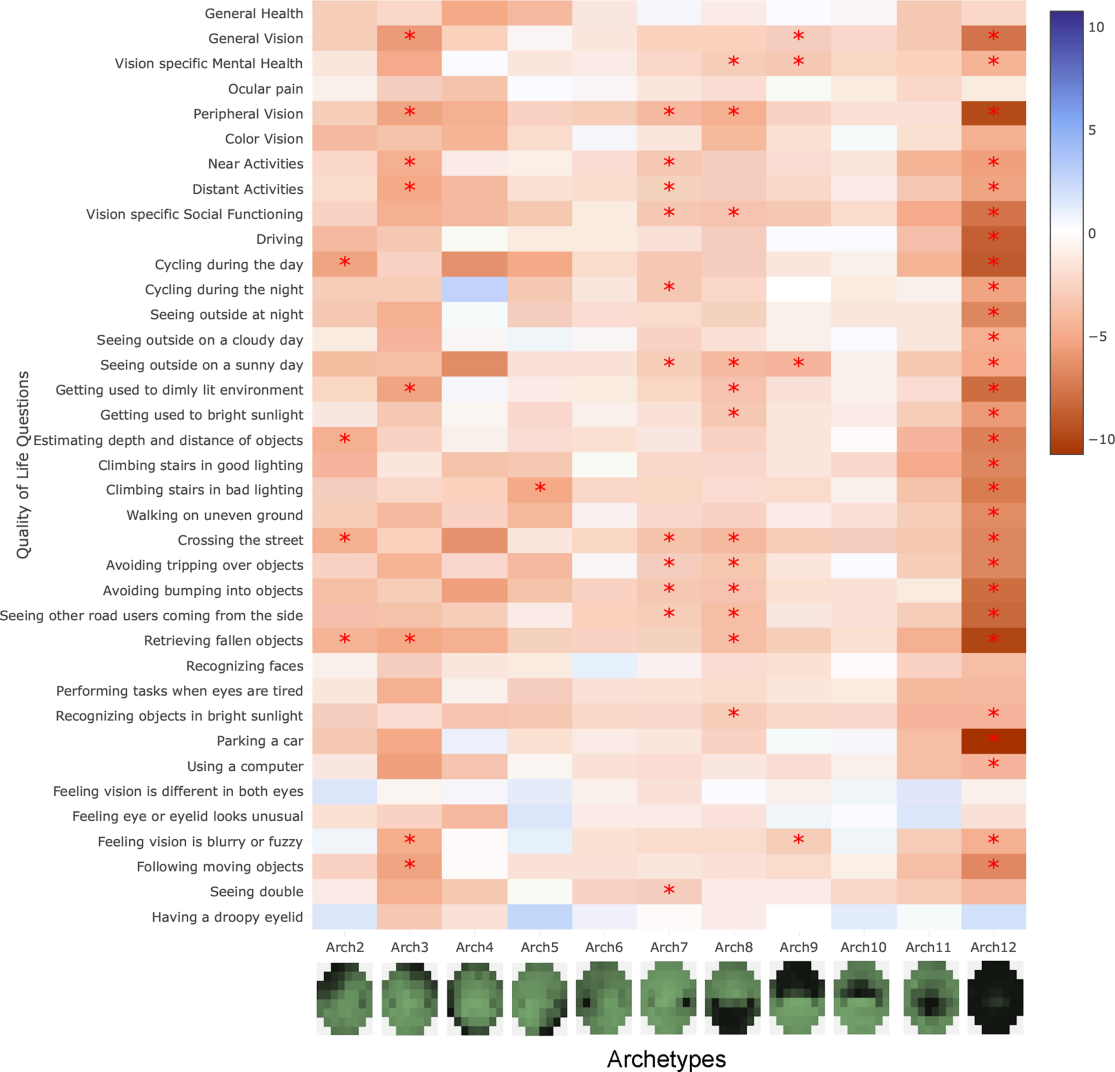
Results of the ordinal regression analysis. Our analysis was adjusted for age, gender, and binocular foveal sensitivity. Each *row* shows the results of the regression for that row's VR-QoL aspect. A positive coefficient indicates an improvement in that aspect of VR-QoL, whereas a negative coefficient indicates a decline. *Asterisks* denote statistically significant associations using Bonferroni adjusted thresholds.

The results of logistic regression analysis adjusted for age, gender, and binocular foveal sensitivity are shown in Figure 5. For the corresponding effect sizes with confidence interval, see the supplementary interactive heatmap. Archetype 12 had a significant correlation with 29 of 37 aspects of VR-QoL measured. Archetypes 4, 6, 10, and 11 had no significant relationship with any of the measured VR-QoL aspects. In the same analysis without adjusting for foveal sensitivity, archetype 11 is significantly associated with 20 of 37 QoL aspects. Regression results without adjusting for foveal sensitivity are provided as Supplementary Material. We did not find a significant impact of archetypes on general health, ocular pain, recognizing faces, feeling that vision is different between eyes, feeling eye or eyelid look unusual, and having a droopy eyelid. Problems with peripheral vision, on the contrary, were significantly associated with six different archetypes. Interestingly, archetype 8, which corresponds to defects in the inferior hemifield, was associated with more aspects of VR-QoL than archetype 9, which corresponds to defects in the superior hemifield.

**Figure 6.**
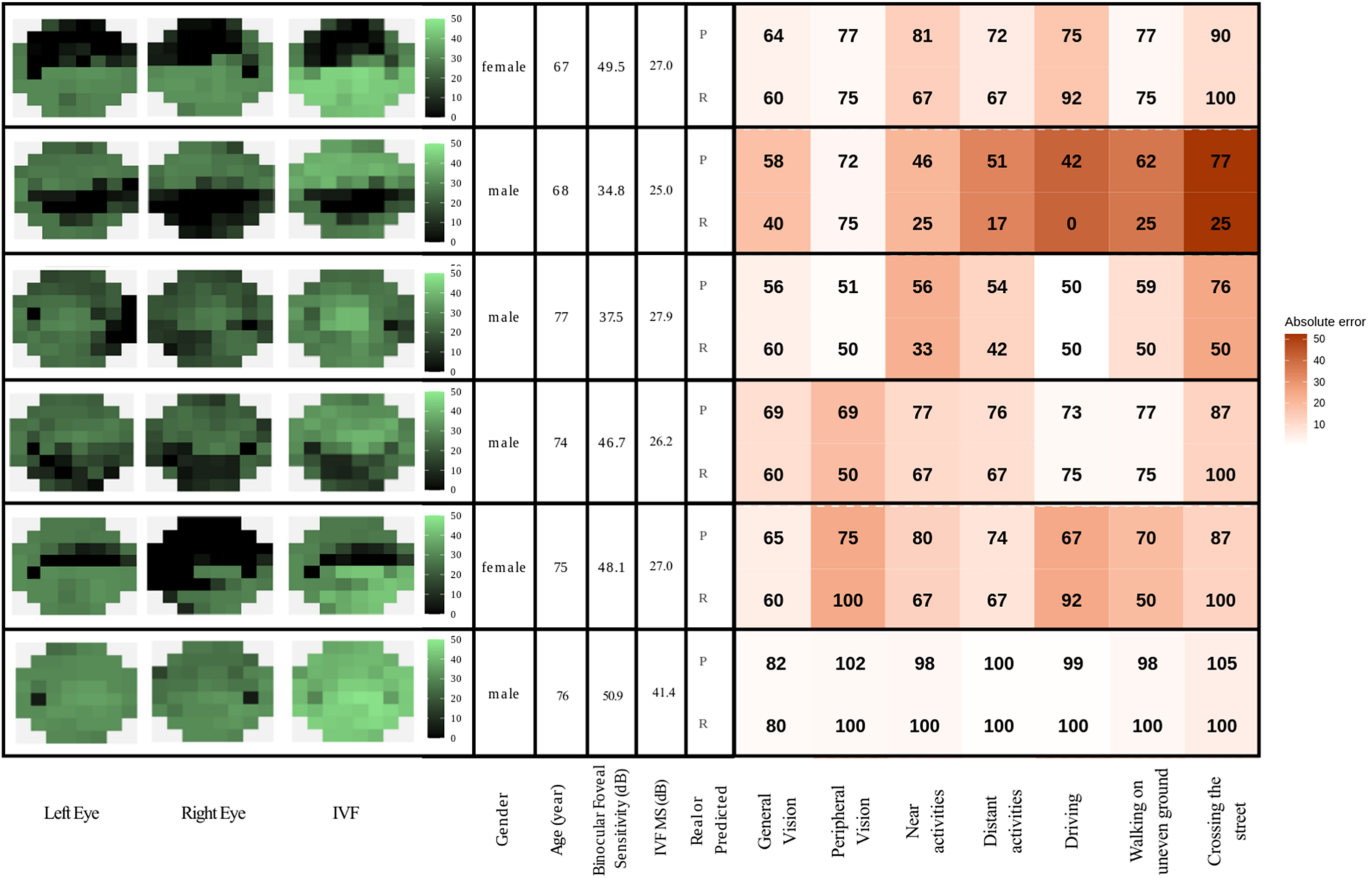
Predicted (P) and real (R) questionnaire scores for six selected patients alongside the left eye, the right eye, and IVF visual field maps. Higher scores correspond to a better quality of life.

To illustrate how different VF patterns relate to different aspects of VR-QoL, we selected some patients with similar age (67 to 77 years), IVF MS values (25 to 28 dB), and binocular foveal sensitivity (35 to 50 dB). Figure 6 presents their predicted and real VR-QoL scores. The last case, with a near normal IVF, is included for contrast. Despite some substantial absolute errors, the examples show that the archetype-based model captures meaningful differences in patient-reported function even when global disease severity is similar.

## Discussion

We identified 12 distinct BVF loss archetypes. These archetypes reflect common patterns of VF loss in glaucoma and were significantly associated with multiple aspects of VR-QoL. Widespread VF loss (archetype 12) has a significant impact on VR-QoL. In contrast, inferior paracentral loss (archetype 11) did not show an independent effect in models adjusted for foveal sensitivity. However, when foveal sensitivity was not included as a covariate, archetype 11 became highly associated with several aspects of VR-QoL. Additionally, vision loss in the inferior hemifield (archetype 8) tends to have a greater impact on daily life than loss in the superior hemifield (archetype 9).

To date, to the best of our knowledge, only one study has explored patterns of BVF defects in glaucoma. Hu et al., using the same UK-NOD dataset, identified, with factor analysis, the most common patterns of BVF defects in glaucoma. They reported 12 factors that closely align with our findings using archetypal analysis. However, they did not examine the relationship between these factors and different aspects of VR-QoL.^34^ With archetypal analysis, we expressed a patient's VF as a weighted combination of clinically interpretable VF loss patterns. Subsequently, multivariable analysis allowed us to relate specific VF loss patterns to specific aspects of VR-QoL, providing insights that are not accessible through severity measures like mean deviation alone.

The VFs from the UK database, drawn from regional NHS glaucoma clinics, had, on average, a slightly better mean deviation and were from older subjects, compared with the VFs from the Netherlands database, which were from a tertiary center outpatient clinic. This was also reflected in the higher prevalence of the archetypes 8 and 9, which correspond to more severe diffuse defects. Apart from this difference, the overall distribution of archetypes was highly similar between the two datasets. This supports the generalizability of the archetypical analysis across different data sets.

Several studies tried to elucidate the relationship between the location of VF loss and its impact on VR-QoL in glaucoma. Methods used in these studies are diverse and the results are not always aligned. McKean-Cowdin et al. reported that VF loss in the central locations lead to a lower VR-QoL as assessed by the NEI-VFQ-25.^8^ Their results are in contradiction with the findings of Peters et al., who found that VF loss in the central four test locations of the HFA (24-2/30-2) are not independently associated with a lower VR-QoL.^9^ Results from the current study and two earlier studies are both in line with Peters et al., confirming that, when adjusted for visual acuity or foveal sensitivity, VF loss in the central four test locations does not independently impact the VR-QoL.^35^^,^^36^ Another finding in our study was the higher impact of VF defects in the right blindspot (archetype 7) on VR-QoL compared to the left blindspot (archetype 6). Interestingly, a study in Japan showed that VF defects in the left blindspot are the second most important variable for all tasks and general VR-QoL after worse-eye visual acuity.^6^ Considering that our results are based on patients living in the Netherlands and a right blindspot defect in the BVF likely indicates a much worse left eye VF, unable to compensate for the right eye blindspot and vice versa, this discrepancy could tentatively be linked to left/right differences between Japan and The Netherlands. For example, the traffic in Japan is left-sided and reading is from right to left, whereas in the Netherlands the traffic is right-sided and people read from left to right.

Several studies have shown that VF loss in the lower hemifield has a higher impact on VR-QoL of patients with glaucoma compared to the upper hemifield.^4^^,^^6^^,^^7^^,^^11^^–^^13^ This is in line with our findings that archetype 8, which is VF loss in the lower hemifield, was negatively associated with more aspects of VR-QoL compared to archetype 9, which is VF loss in the upper hemifield. Moreover, archetype 8 was associated with problems with mobility, which is in line with reports suggesting that patients with glaucoma with a VF loss in the inferior hemifield have a slower walking speed and a higher rate of falls.^37^^,^^38^

Our study identified a significant and strong relationship between global VF loss (represented by archetype 12) and driving performance. Whereas the impact of VF defects on driving is well-established,^39^^–^^41^ the specific influence of defect location remains unresolved – previous studies have resulted in conflicting insights regarding the distinct impact of superior versus inferior defects on driving. Some studies suggested the lower hemifield is more critical, possibly affecting tasks such as checking side-view mirrors, monitoring the speedometer, and maintaining road awareness,^5^^,^^42^ whereas other studies indicated the upper hemifield is more significant, highlighting superior altitudinal defects’ pronounced effect on hazard detection.^4^^,^^14^ Neither inferior nor superior VF defects individually showed significance in our study, aligning with a recent systematic review that highlighted insufficient evidence when comparing the impact of superior versus inferior defects in driving.^42^ Importantly, archetype 11 (inferior paracentral loss) was not associated with driving in the primary analysis, but became highly significant when the analysis was not adjusted for foveal sensitivity. This entanglement between foveal sensitivity and paracentral VF loss in VR-QoL research has been reported earlier.^36^

Identification of distinct BVF loss archetypes enables clinicians to better predict the functional consequences of glaucomatous damage. Understanding which patterns of VF loss are most detrimental to VR-QoL can help to prioritize treatment and management strategies aimed at preserving or improving (via targeted rehabilitation) patients’ QoL. Moreover, the strong associations between specific archetypes and various VR-QoL aspects highlight the need for a personalized approach to glaucoma management – some patients may suffer more from a certain pattern of VF loss than others, because they have other needs. Tailoring interventions based on the patient’s specific pattern of VF loss may optimize outcomes and enhance VR-QoL. For example, patients with inferior hemifield defects, which are strongly associated with mobility challenges, such as seeing other road users coming from the side, crossing the street, or avoiding tripping over or bumping into objects, may benefit from targeted interventions such as mobility training or orientation aids to mitigate the impact on daily activities.

We demonstrated the use of an archetypal model to predict VR-QoL in individual patients (see Fig. 6). However, these predictions should be interpreted with caution because this prediction model was, unlike the archetypes themselves, trained and tested on the same dataset, which inflates its apparent performance. Importantly, the main objective of our study was to investigate associations, not to generate accurate predictions of VR-QoL. In addition, patient-reported outcome measures (PROMs) such as VR-QoL questionnaires show considerable interpersonal and intrapersonal variability, which makes accurate prediction of PROM-based outcomes inherently difficult.^43^ After all, two patients with the same visual function may experience a different VR-QoL if their visual demands differ. For that reason, performance-based assessments of functional vision, such as the Assessment of Disability Related to Vision (ADREV), offer a more objective alternative.^44^ These tests measure actual task performance and have been shown to correlate more consistently with clinical indices, including visual acuity, contrast sensitivity, and VF loss compared with PROM instruments.^45^ In several patient groups, ADREV demonstrated stronger associations with clinical measurements than the NEI-VFQ-25, indicating greater stability and lower subjective noise.^46^^,^^47^ On the other hand, PROMs better reflect what patients actually experience.

A notable strength of our study is the novel methodological approach, as it is the first to apply archetypal analysis to BVF loss in glaucoma, enabling a nuanced characterization of spatial patterns of VF defects. Furthermore, the archetypal analysis was conducted on an extensive dataset comprising over 7000 patients with glaucoma, which provided robust statistical power and increased the reliability of our findings. Additionally, the associations between identified archetypes and VR-QoL were evaluated using another independent dataset. However, our study has some limitations as well. First, the cross-sectional nature of the analysis limits our ability to draw causal inferences between BVF loss archetypes and VR-QoL. Second, although we aimed to include only patients with glaucoma and exclude those with neurological defects, our method for detecting neurological VF defects was effective only for post-chiasmatic defects. Consequently, the patient population used for the archetype identification may have been polluted with individuals with chiasmatic or pre-chiasmatic, non-glaucomatous VF defects. Moreover, GLGS participants were required to complete a questionnaire, which inevitably limits the sample to patients willing to provide this information. This could have introduced bias, but not necessarily in one direction. Although it is possible that patients with better functioning and higher QoL were more inclined to participate, it is equally plausible that patients more affected by their disease and motivated to share their experience were over-represented. Another limitation of our study is that the functional vision outcomes were derived from questionnaires. These are subject to perception, mood, and response style, which increases intra- and interindividual variability. In contrast, performance-based assessments use standardized tasks and objective scoring and likely provide a more stable estimate of functional capacity.^44^^,^^47^ On the other hand, they may reflect less accurately what patients actually experience. Last, archetypal analysis may fail to capture specific patterns of VF loss, especially if they are relatively rare.

This study identified distinct BVF loss archetypes and their associations with various VR-QoL domains and can be a starting point for incorporating the location of a VF defect in a more personalized management of patients with glaucoma. Our findings underscore the importance of considering both the extent and pattern of visual field loss in clinical decision making to enhance patient outcomes and QoL. Future research on this topic could benefit from a longitudinal study design, which could track changes in BVF loss archetypes and VR-QoL over time, helping to establish causal links. Furthermore, using performance-based assessments like ADREV instead of questionnaires helps in mapping patients’ functional capacity more accurately.^48^

## Supplementary Material

Supplement 1
