# Supplementary material for: Archetypes of Binocular Visual Field Loss and Their Impact on Vision-Related Quality of Life in Glaucoma Patients
Source: Invest Ophthalmol Vis Sci. 2026 Mar 12;67(3):28. doi: 10.1167/iovs.67.3.28 (PMC12988670; doi:10.1167/iovs.67.3.28)
Supplement: Supplement 1 [file iovs-67-3-28_s001.zip › Supplementary Questionnaire.docx (1).pdf]

Beginning of questionnaire

Date: _______-_______-________

Age: _________________ years ▢ Female ▢ Male

PART 1 - GENERAL HEALTH AND VISION

1. In general, would you say your overall health is:

▢ Excellent

▢ Very good

▢ Good

▢ Fair

▢ Poor

2. At the present time, would you say your eyesight using both eyes (with glasses or contact lenses, if you wear them) is excellent, good, fair, poor, or very poor or are you completely blind?

▢ Excellent

▢ Good

▢ Fair

▢ Poor

▢ Very poor

▢ Completely blind

3. How much of the time do you worry about your eyesight?

▢ None of the time

▢ A little of the time

▢ Some of the time

▢ Most of the time

▢ All of the time

4. How much pain or discomfort have you experienced in and around your eyes (e.g., burning, itching, or pain)? Would you say it is:

▢ None

▢ Mild

▢ Moderate

▢ Severe

▢ Very severe

PART 2 - DIFFICULTY IN CARRYING OUT ACTIVITIES

The next questions are about the difficulty, if any, you have doing certain activities wearing your glasses or contact lenses if you use them for that activity.

5. How much difficulty do you have reading ordinary print in newspapers? Would you say you have:

▢ No difficulty

▢ A little difficulty

▢ Moderate difficulty

▢ Extreme difficulty

▢ Stopped doing this because of your eyesight

▢ Stopped doing this for other reasons or not interested in doing this

6. Do you have difficulty with activities or hobbies that require close vision, such as cooking, sewing, repairing things around the house, or using hand tools?

▢ No difficulty

▢ A little difficulty

▢ Moderate difficulty

▢ Extreme difficulty

▢ Stopped doing this because of your eyesight

▢ Stopped doing this for other reasons or not interested in doing this

7. Do you have trouble, because of your eyesight, finding something on a full shelf?

- ▢ No difficulty
- ▢ A little difficulty
- ▢ Moderate difficulty
- ▢ Extreme difficulty
- ▢ Stopped doing this because of your eyesight
- ▢ Stopped doing this for other reasons or not interested in doing this

8. Do you have trouble reading street signs or the names of stores?

- ▢ No difficulty
- ▢ A little difficulty
- ▢ Moderate difficulty
- ▢ Extreme difficulty
- ▢ Stopped doing this because of your eyesight
- ▢ Stopped doing this for other reasons or not interested in doing this

9. Do you have difficulty, because of your eyesight, stepping down a step, stair or curb in poor lighting or at night?

- ▢ No difficulty
- ▢ A little difficulty
- ▢ Moderate difficulty
- ▢ Extreme difficulty
- ▢ Stopped doing this because of your eyesight
- ▢ Stopped doing this for other reasons or not interested in doing this

10. Do you have trouble, because of your eyesight, noticing things off to the side as you walk past them?

- ▢ No difficulty
- ▢ A little difficulty
- ▢ Moderate difficulty
- ▢ Extreme difficulty
- ▢ Stopped doing this because of your eyesight
- ▢ Stopped doing this for other reasons or not interested in doing this

11. Do you have trouble, because of your eyesight, seeing how people react to what you say?

- ▢ No difficulty
- ▢ A little difficulty
- ▢ Moderate difficulty
- ▢ Extreme difficulty
- ▢ Stopped doing this because of your eyesight
- ▢ Stopped doing this for other reasons or not interested in doing this

12. Do you have trouble, because of your eyesight, picking out and combining your own clothes?

- ▢ No difficulty
- ▢ A little difficulty
- ▢ Moderate difficulty
- ▢ Extreme difficulty
- ▢ Stopped doing this because of your eyesight
- ▢ Stopped doing this for other reasons or not interested in doing this

13. Because of your eyesight, do you have trouble visiting people, going to parties or restaurants?

- ▢ No difficulty
- ▢ A little difficulty
- ▢ Moderate difficulty
- ▢ Extreme difficulty
- ▢ Stopped doing this because of your eyesight
- ▢ Stopped doing this for other reasons or not interested in doing this

14. Do you have difficulty, because of your eyesight, seeing cinema films, theatre or sporting events?

- ▢ No difficulty
- ▢ A little difficulty
- ▢ Moderate difficulty
- ▢ Extreme difficulty
- ▢ Stopped doing this because of your eyesight
- ▢ Stopped doing this for other reasons or not interested in doing this

15. Are you currently driving, at least once in a while?

- ▢ Yes (Skip to question 15c)
- ▢ No

15a. IF NO: Have you never driven a car or have you given up driving?

- ▢ Never drove (Skip to question 17)
- ▢ Gave up

15b. IF YOU GAVE UP DRIVING: Was that <u>mainly because of your eyesight</u>, <u>mainly for some other reason</u>, or because of <u>both your eyesight and other reasons</u>?

▢ Mainly eyesight (Skip to question 17)
▢ Mainly other reasons (Skip to question 17)
▢ Both eyesight and other reasons (Skip to question 17)

15c. IF CURRENTLY DRIVING: How much difficulty do you have <u>driving during the daytime in familiar places</u>? Would you say you have:

▢ No difficulty
▢ A little difficulty
▢ Moderate difficulty
▢ Extreme difficulty

16. How much difficulty do you have <u>driving at night</u>? Would you say you have:

▢ No difficulty
▢ A little difficulty
▢ Moderate difficulty
▢ Extreme difficulty
▢ Stopped doing this because of your eyesight
▢ Stopped doing this for other reasons or not interested in doing this

16a. How much difficulty do you have <u>driving in difficult conditions, such as in bad weather, during rush hour, on the freeway, or in city traffic</u>? Would you say you have:

▢ No difficulty
▢ A little difficulty
▢ Moderate difficulty
▢ Extreme difficulty
▢ Stopped doing this because of your eyesight
▢ Stopped doing this for other reasons or not interested in doing this

If you have glasses/contact lenses, please answer the questions as if you were wearing them. <u>Because of your eyesight</u>, do you have any trouble with...	No difficulty	A little difficulty	Moderate difficulty	Extreme difficulty	Stopped doing this because of your eyesight	Stopped doing this for other reasons or not interested in doing this
17. cycling during the day, in an environment that is familiar to you	▢	▢	▢	▢	◯	◯
18. walking or cycling at night, on a non-lit outdoor road	▢	▢	▢	▢	◯	◯
19. see outside at night without moonlight	▢	▢	▢	▢	◯	◯
20. seeing outside on a cloudy day	▢	▢	▢	▢	◯	◯
21. see outside on a sunny day	▢	▢	▢	▢	◯	◯
22. get used to a dimly-lit environment, if you are coming from a well-lit environment	▢	▢	▢	▢	◯	◯
23. getting used to bright sunlight, if you are coming from an environment with less light	▢	▢	▢	▢	◯	◯
24. estimating the depth and distance of objects (e.g. when grasping a glass)	▢	▢	▢	▢	◯	◯
25. climbing stairs with good lighting	▢	▢	▢	▢	◯	◯
26. climbing stairs in poor lighting conditions	▢	▢	▢	▢	◯	◯
27. seeing where you are walking on uneven ground, for example on cobblestones	▢	▢	▢	▢	◯	◯
28. crossing the street	▢	▢	▢	▢	◯	◯
29. avoiding tripping over objects	▢	▢	▢	▢	◯	◯
30. avoidance of bumping into objects	▢	▢	▢	▢	◯	◯
31. seeing other road users coming from the side	▢	▢	▢	▢	◯	◯
32. recovery of fallen objects	▢	▢	▢	▢	◯	◯
33. recognising the faces of people you meet	▢	▢	▢	▢	◯	◯

34. How much difficulty do you have performing tasks when your eyes are <u>tired</u>?

- ▢ No difficulty
- ▢ A little difficulty
- ▢ Moderate difficulty
- ▢ Extreme difficulty

35. Because of your vision, how much difficulty do you have identifying objects or performing tasks in bright sunlight?

- ▢ No difficulty
- ▢ A little difficulty
- ▢ Moderate difficulty
- ▢ Extreme difficulty

36. Because of your vision, how much difficulty do you have parking a car?

- ▢ No difficulty
- ▢ A little difficulty
- ▢ Moderate difficulty
- ▢ Extreme difficulty
- ▢ Stopped doing this because of your eyesight
- ▢ Stopped doing this for other reasons or not interested in doing this

37. Because of your vision, how much difficulty do you have using a computer?

- ▢ No difficulty
- ▢ A little difficulty
- ▢ Moderate difficulty
- ▢ Extreme difficulty
- ▢ Stopped doing this because of your eyesight
- ▢ Stopped doing this for other reasons or not interested in doing this

38. I have a feeling that my two eyes see differently, even with correction (glasses or contact lenses).

▢ Definitely true

▢ Mostly true

▢ Not sure

▢ Mostly false

▢ Definitely false

39. I have a feeling that my eye or eyelid appearance is unusual.

▢ Definitely true

▢ Mostly true

▢ Not sure

▢ Mostly false

▢ Definitely false

40. My vision is blurry, not clear, or “fuzzy.”.

▢ All of the time

▢ Most of the time

▢ Some of the time

▢ A little of the time

▢ None of the time

41. I have trouble focusing on or following moving objects.

▢ All of the time

▢ Most of the time

▢ Some of the time

▢ A little of the time

▢ None of the time

42. I have double vision with both eyes open that is not present when either eye is covered.

- ▢ All of the time
- ▢ Most of the time
- ▢ Some of the time
- ▢ A little of the time
- ▢ None of the time

43. My eyelid(s) droop.

- ▢ All of the time
- ▢ Most of the time
- ▢ Some of the time
- ▢ A little of the time
- ▢ None of the time

OPEN QUESTION

44. Are there other tasks or activities that cause you trouble because of your eyesight, that have not been covered by this questionnaire? Think for instance of specific sports or hobbies, or a profession you had to give up.

OTHER REMARKS OR SUGGESTIONS
