# Supplementary figures and images for: Archetypes of Binocular Visual Field Loss and Their Impact on Vision-Related Quality of Life in Glaucoma Patients
Source: Invest Ophthalmol Vis Sci. 2026 Mar 12;67(3):28. doi: 10.1167/iovs.67.3.28 (PMC12988670; doi:10.1167/iovs.67.3.28)

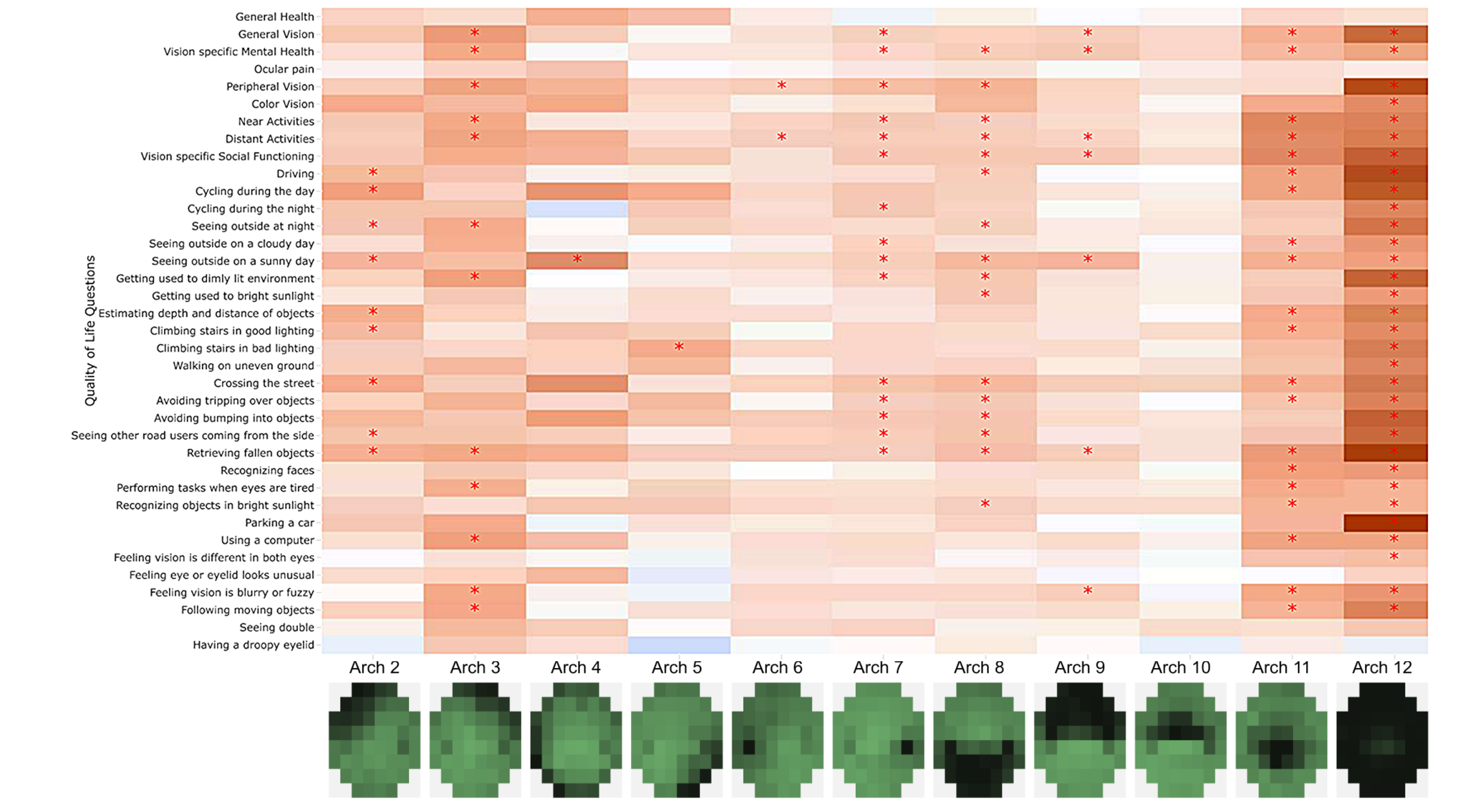

Supplement: Supplement 1 [file iovs-67-3-28_s001.zip › Supplementary Figure 1 (1).PNG]
